# An Intermediate Concentration of Calcium with Antioxidant Supplement in Culture Medium Enhances Proliferation and Decreases the Aging of Bone Marrow Mesenchymal Stem Cells

**DOI:** 10.3390/ijms22042095

**Published:** 2021-02-20

**Authors:** Chung-Da Yang, Shu-Chun Chuang, Tsung-Lin Cheng, Mon-Juan Lee, Hui-Ting Chen, Sung-Yen Lin, Hsuan-Ti Huang, Cheng-Jung Ho, Yi-Shan Lin, Lin Kang, Mei-Ling Ho, Je-Ken Chang, Chung-Hwan Chen

**Affiliations:** 1 Graduate Institute of Animal Vaccine Technology, College of Veterinary Medicine, National Pingtung University of Science and Technology, Pingtung 912301, Taiwan; cdyang@mail.npust.edu.tw; 2 Orthopaedic Research Center, Kaohsiung Medical University, Kaohsiung 80701, Taiwan; f86225016@ntu.edu.tw (S.-C.C.); junglecc@kmu.edu.tw (T.-L.C.); sungyenlin@kmu.edu.tw (S.-Y.L.); hthuang@kmu.edu.tw (H.-T.H.); u105801010@kmu.edu.tw (C.-J.H.); r950084@kmu.edu.tw (Y.-S.L.); homelin@cc.kmu.edu.tw (M.-L.H.); 3 Regeneration Medicine and Cell Therapy Research Center, Kaohsiung Medical University, Kaohsiung 80701, Taiwan; 4 Musculoskeletal Regeneration Research Center, Kaohsiung Medical University, Kaohsiung 80701, Taiwan; 5 Department of Physiology, College of Medicine, Kaohsiung Medical University, Kaohsiung 80701, Taiwan; 6 Department of Bioscience Technology, Chang Jung Christian University, Tainan 71101, Taiwan; mjlee@mail.cjcu.edu.tw; 7 Innovative Research Center of Medicine, Chang Jung Christian University, Tainan 71101, Taiwan; 8 Faculty of Pharmacy, School of Pharmaceutical Sciences, National Yang-Ming University, Taipei 11221, Taiwan; htchen1969@ym.edu.tw; 9 Department of Fragrance and Cosmetic Science, Kaohsiung Medical University, Kaohsiung 80701, Taiwan; 10 Department of Orthopedics, Kaohsiung Medical University Hospital, Kaohsiung Medical University, Kaohsiung 80701, Taiwan; 11 Departments of Orthopedics, College of Medicine, Kaohsiung Medical University, Kaohsiung 80701, Taiwan; 12 Department of Orthopedics, Kaohsiung Municipal Ta-Tung Hospital, Kaohsiung 80145, Taiwan; 13 Graduate Institute of Medicine, College of Medicine, Kaohsiung Medical University, Kaohsiung 80701, Taiwan; 14 Department of Obstetrics and Gynecology, National Cheng Kung University Hospital, College of Medicine, National Cheng Kung University, Tainan 701, Taiwan; kanglin@mail.ncku.edu.tw; 15 Department of Marine Biotechnology and Resources, National Sun Yat-Sen University, Kaohsiung 80424, Taiwan; 16 Department of Medical Research, Kaohsiung Medical University Hospital, Kaohsiung Medical University, Kaohsiung 80701, Taiwan; 17 Institute of Medical Science and Technology, National Sun Yat-Sen University, Kaohsiung 80420, Taiwan; 18 Department of Healthcare Administration and Medical Informatics, Kaohsiung Medical University, Kaohsiung 80701, Taiwan

**Keywords:** culture medium, cell aging, cell proliferation, cell differentiation, senescence

## Abstract

Human bone marrow stem cells (HBMSCs) are isolated from the bone marrow. Stem cells can self-renew and differentiate into various types of cells. They are able to regenerate kinds of tissue that are potentially used for tissue engineering. To maintain and expand these cells under culture conditions is difficult—they are easily triggered for differentiation or death. In this study, we describe a new culture formula to culture isolated HBMSCs. This new formula was modified from NCDB 153, a medium with low calcium, supplied with 5% FBS, extra growth factor added to it, and supplemented with N-acetyl-L-cysteine and L-ascorbic acid-2-phosphate to maintain the cells in a steady stage. The cells retain these characteristics as primarily isolated HBMSCs. Moreover, our new formula keeps HBMSCs with high proliferation rate and multiple linage differentiation ability, such as osteoblastogenesis, chondrogenesis, and adipogenesis. It also retains HBMSCs with stable chromosome, DNA, telomere length, and telomerase activity, even after long-term culture. Senescence can be minimized under this new formulation and carcinogenesis of stem cells can also be prevented. These modifications greatly enhance the survival rate, growth rate, and basal characteristics of isolated HBMSCs, which will be very helpful in stem cell research.

## 1. Introduction

Mesenchymal cells (MSCs) are adult multipotent stem cells that can be isolated from various tissues such as bone marrow, adipose tissue, fetal membranes of term placenta, dental pulp, and ovarian follicular liquid. MSCs can differentiate into osteoblasts, chondrocytes, and adipocytes. Because the use of human embryonic stem cells is ethically controversial, more studies has focused on MSC for clinical applications [1,2,3,4]. Bone marrow, a soft blood-forming living tissue, is composed of bone cavities that contain fat, immature and mature blood cells, hematopoietic stem cells, and non-hematopoietic stem cells. Non-hematopoietic stem cells are located in the bone marrow stoma. Bone marrow stroma cells—a mixed cell population—contain MSCs, in which stem cells generate bone, cartilage, fat, fibrous connective tissue, and the reticular network that supports blood cell formation [5,6]. Human bone mesenchymal stem cells (HBMSCs) belong to adult stem cells, like all stem cells, sharing at least two characteristics. First, they can make identical copies of themselves for long periods of time; this ability to proliferate is referred to as long-term self-renewal. Second, they can give rise to mature cell types that have characteristic morphologies (shapes) and specialized functions [7,8].

Adult stem cells are able to reproduce itself for long periods throughout the life of the organism. Multipotential mesenchymal stem cells were known as early as 1968 [8,9] through the work of Friedenstein and his coworkers, who established that cells that are adherent, clonogenic, nonphagocytic, and fibroblastic in habit (defined as colony-forming units–fibroblastic; CFU-Fs) can be isolated from the bone marrow stroma of postnatal organisms. From now on, human mesenchymal stem cells are so-called multipotential bone marrow cells that can be expanded ex vivo; they usually divide to generate progenitor or precursor cells, which then differentiate or develop into “mature” cell types that have characteristic shapes and specialized functions [5], such as cartilage, bone, and fat [10,11,12].

It has also been suggested that skeletal tissue, muscle, and even nervous tissue can be regenerated from stem cell populations [7,13], which could be engineered to replace or repair a defective gene. Stem cells have self-renewal properties and the ability to regenerate tissues; transgenic stem cells, thus, could have a long-lasting effect. Osteoporosis (OP) and osteoarthritis (OA) are two common diseases in orthopedics [4,14]. Stem cells are useful not only for the treatment of OP or OA, but also for trauma, cancer, or malformations [2]. There is no current medication that can cure OP or OA. Some medications that can be beneficial for the above diseases include bisphosphonates and non-steroidal anti-inflammatory drugs [15], respectively. Total joint replacement may be required for patients having advanced OA with severe pain.

Isolated HBMSCs are promising as a cell source for a variety of tissue engineering and cell therapy applications [16]. Stem cells have the ability to self-renew, migrate, and engraft within various tissues. On the other hand, if stem cells are modified with genetic engineering to carry specific genes such as bone repair specific gene expressions (e.g., bone morphogenetic protein-2), vessel growth specific gene expression (e.g., vascular endothelial growth factor), or chondrocyte growth specific gene expression (e.g., transforming growth factor-beta), it may enhance the repair. However, the frequency of HBMSCs on human bone marrow is not high [17]. To maintain and expand these cells under culture conditions is difficult [1,18]—they are easily triggered for differentiation or death. Therefore, it is not easy to obtain adequate HBMSCs to perform cell therapy. Accordingly, the formula for efficient culture and growth of adult HBMSCs would be useful and necessary to yield good and ample HBMSCs that are clinically beneficial for a recipient [7]. This new formula could offer a suitable condition to amplify HBMSCs and keep them in a steady stage; if necessary, these cells can then easily be triggered to differentiate. By using a new formula for isolating and culturing HBMSCs, we can repopulate them either during normal life or at least under circumstances of tissue repair [19]. Primary cultures of HBMSCs were established by bone marrow aspirates from iliac crest and isolated by 70% percoll gradients as per a previous report [20]. These cells are undifferentiated cells with capacity of unlimited or prolonged self-renewal and the ability to give rise to differentiated descendants and can be stimulated to differentiate into bone, cartilage, muscle, marrow stroma, tendon, fat, and a variety of other connective tissues [21,22,23].

## 2. Results

### 2.1. Isolation of Human Bone Marrow Stem Cells

**Figure 1:**
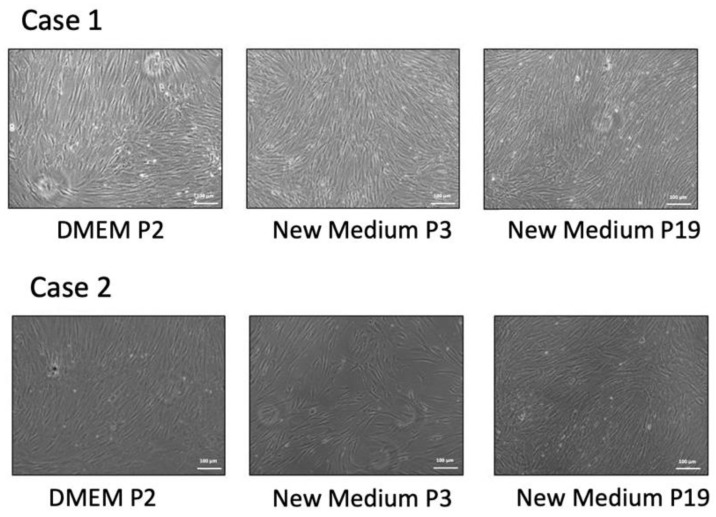
The cell morphology of HBMSC cultured in traditional medium (DMEM) and new formula medium (BK medium). The morphology of the cells cultured in the traditional media was similar to that cultured in the new formula medium.

HBMSCs were isolated by 70% percoll gradients. We compared the morphology of HBMSCs cultured traditionally with this new formula (BK media). The traditional culture media is DMEM apply 10% FBS plus 1% vitamin C and antibiotics (100 units of penicillin G per milliliter and 100 micrograms of streptomycin per milliliter). Isolated HBMSCs were isolated and cultured in the traditional media until passage 2, then passage 1 × 10^5^ cells into a 100-mm dish, and subsequently applied with the new formula with media changed every other day. The morphology of each passage was checked. The morphology of cells cultured in the traditional media was similar to that cultured in the new formula medium. (Figure 1).

### 2.2. Anchorage Independent Growth

**Figure 2:**
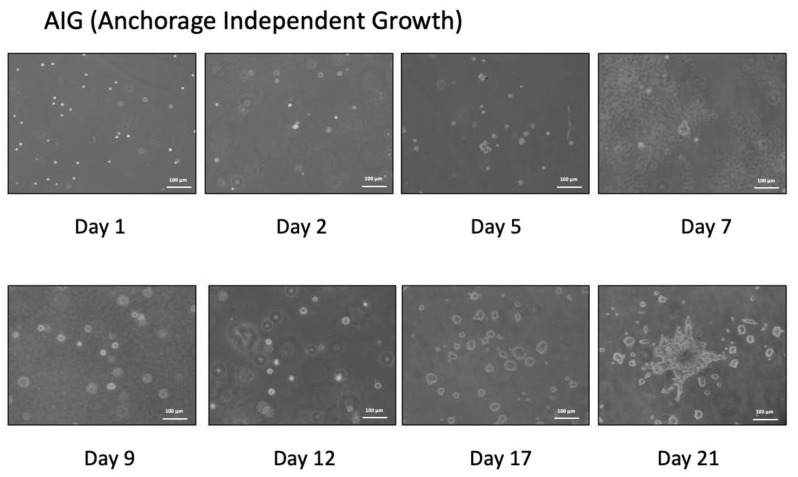
The anchorage independent growth of HBMSC cultured in BK medium. Cells can grow and have surprisingly high frequencies of AIG.

A total of 50,000 cells in 3 mL of 0.33% agarose medium were plated on top of 3 mL of prehardened 0.5% agarosemedium in each of the triplicate dishes (6 cm). Then, 2.5 mL of liquid medium BK media was added; the media was changed every two days. Cells can grow and have surprisingly high frequencies of AIG (62.4% and 55.8%, respectively). All experiments were repeated three times (Figure 2).

### 2.3. Proliferation Potential of Putative Human Bone Marrow Stem Cells

**Figure 3:**
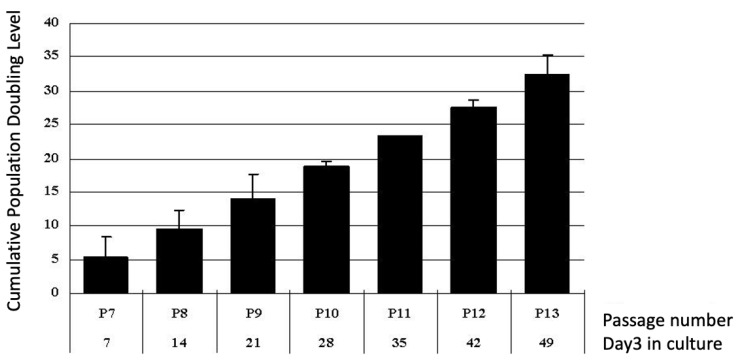
Average accumulative population doubling from three cases was shown. The average population doubling was about 4.2–4.5 folds, in the late stage (e.g., 16th passage) of cultured cell, maintaining similar population doubling. The average population doubling in DMEM were about 2.2–2.7 folds in the late stage (e.g., 7th–8th passage).

To determine the proliferation of cells cultured in BK media, HBMSCs were cultured. Every seven days, cell number was counted and population doubling was checked. Average accumulative population doubling from three cases is shown in Figure 3. The average population doubling was about 4.2–4.5 folds in the late stage (e.g., 13th passage) of the cultured cell, maintaining similar population doubling. The average population doubling in DMEM were about 2.2–2.7 folds in the late stage (e.g., 7th–8th passage) and the cell morphology is flat (data not shown). These results show that HBMSC can proliferate more passages and cells in BK medium than in DMEM medium.

### 2.4. Induction of Multilineage Stem Cell Differentiation

#### 2.4.1. Osteogenic Differentiation

**Figure 4:**
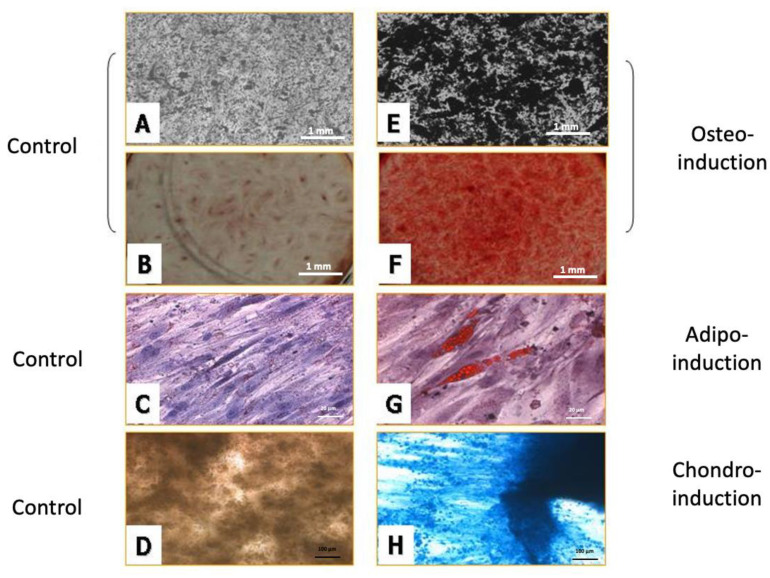
Induction of multilineage stem cell differentiation. Calcification appears as black regions within the cell monolayer in von Kossa stain (**A**,**E**) and red in alizarin S stain (**B**,**F**). Cells develop triglycerol-containing droplets that accumulate the lipid dye Oil Red–O in every passage (**C**,**G**). The cells cultured in the induction showed extensive chondrogenic differentiation while cells cultured in the DMEM showed no chondrogenic differentiation (**D**,**H**).

For osteogenic differentiation, HBMSCs cultured in BM medium or DMEM medium were changed to culture in osteo-induction media for 14 days. After 14 days, ECM calcification was checked by von Kossa stain and alizarin red S stain. Calcification appears as black regions within the cell monolayer in von Kossa staining (Figure 4A,E). In alizarin red S staining (Figure 4B,F), calcification appears as red stain covering the cell surface, and this stain can be isolated and quantified. The results show cells cultured in BK media with more ECM calcification than that in the DMEM medium after osteogenic differentiation. Each stage of cultured HBMSCs in BK medium can be triggered for mineralization.

#### 2.4.2. Adipose Differentiation

After 12 days of adipose differentiation, cells develop triglycerol-containing droplets that accumulate the lipid dye Oil Red–O in every passage [11,20,24]. (Figure 4C, control group and G exp. group). The results show cells cultured in BK media with more lipid than that in the DMEM medium after adipose differentiation. Each stage of cultured HBMSCs in BK medium can be triggered for lipid droplets accumulation.

#### 2.4.3. Chondrogenic Differentiation

For chondrogenic differentiation, HBMSCs cultured in BM medium or DMEM medium were changed to culture in chondrogenic induction media for 14 days. The cells showed a blue color as insoluble complexes with glycosaminoglycans. The results showed cells cultured in BK media had more glycosaminoglycans than that in the DMEM medium after chondrogenic differentiation. Each stage of cultured HBMSCs in BK medium can be triggered for glycosaminoglycan accumulation.

### 2.5. Flow Cytometry and Cell Surface Marker for Stem Cell Specificity

**Figure 5:**
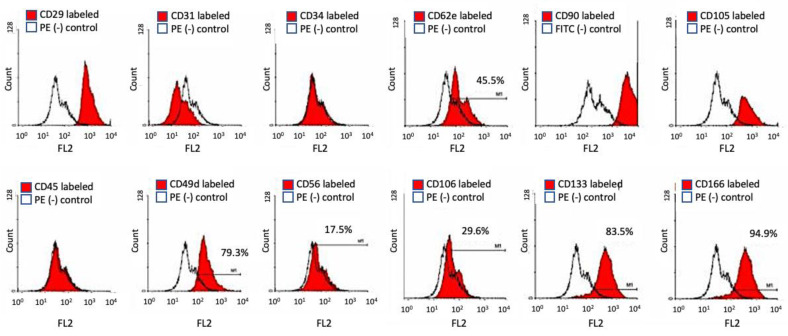
The CD markers expressed on HBMSCs. HBMSCs express CD29, CD90, CD105, CD106, and CD166 and lack expression of the hematopoietic lineage markers CD31, CD34, CD45, CD49D, CD56, and CD106.

HBMSCs of passage 3, 7, 9, 13, 17, and 19 were cultured and labeled with specific cell surface CD markers. HBMSCs express CD29, CD44, CD73, CD90, CD105, and CD106; and lack expression of the hematopoietic lineage markers c-kit, CD14, CD11b, CD31, CD34, CD45, CD49D, CD56 CD62e, CD19, CD79α, CD106, CD133, and human leukocyte antigen (HLA)-DR [25]. The results showed that HBMSCs in the 6th, 13th, and 15th passage expressed similar CD markers, identical to those in previous studies. (Figure 5 and Table 1).

### 2.6. Array Comparative Genomic Hybridization (Array-CGH)

**Figure 6:**
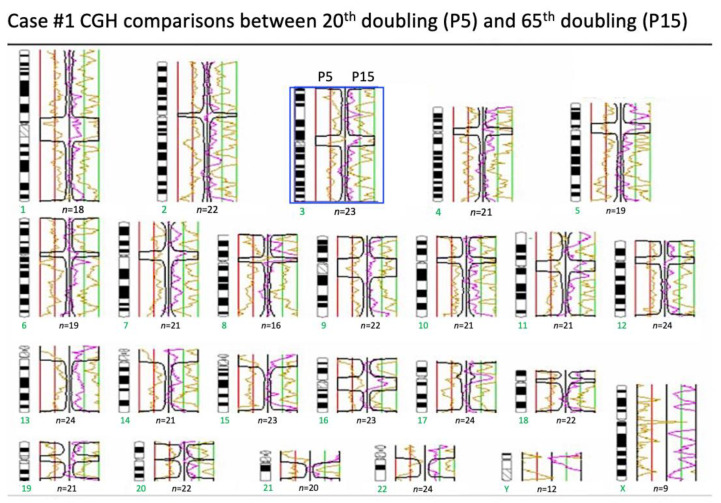
Array comparative genomic hybridization (array-CGH). There was no significant chromosomal change by GCH array in a confidential interval of 99.5% between the 5th (P5) and 15th (P15) passages. The green number meant the chromosome number, X meant X chromosome and Y meant Y chromosome. The blue box showed an example that orange side was the CGH for P5 and the green side was the CGH for P15. The black wave lines showed that the chromosomes were very similar.

On analyzing the 5th passage and the 15th passage of HBMSC cultured in this new formula, no significant chromosomal change was found by GCH array in a confidential interval of 99.5% (Figure 6). This means that even after long-term culture, the stability of chromosomes could be maintained by this new formula.

### 2.7. DNA Damage Protein 53BP1 Immunostaining

**Figure 7:**
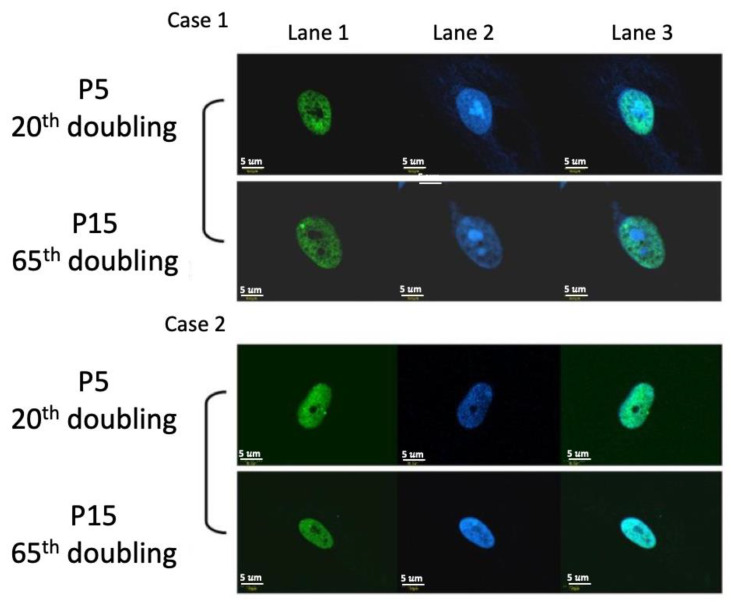
Immunostaining of 53BP1. The figure showed 2 cases of HBMSCs in P5 and P15. The green color (Lane 1) showed the positive staining of anti-53BP1 and the blue color (Lane 2) showed the staining of DAPI. The Lane 3 showed the merge image of anti-53BP1 and DAPI.

In the assay of DNA damage, we use 53BP1 damage protein to monitor the senescence of cells by the accumulation of 53BP1. Under the culture of this new formula, there was no obvious accumulation of 53BP1 at the 5th and 15th passages (Figure 7). Therefore, this new formula can maintain DNA stability.

### 2.8. Telomerase Activity Assay

**Figure 8:**
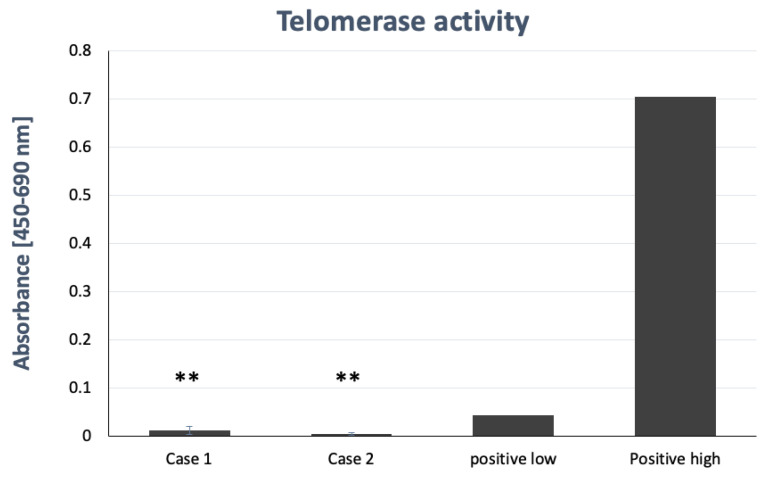
Telomerase activity assay of HBMSCs. The telomerase activity of HBMSCs were very low. A positive low pointed to the control group with low telomerase and positive high to the control group with high telomerase. (** *p* < 0.01 control compared with positive high).

Previous studies have revealed that HBMSCs did not express high activity. Long-term culture of stem cells sometimes led to carcinogenesis by elevated activity of telomerase. Under the culture of this new formula, the telomerase activity of HBMSCs at the 9th passage was still very low (Figure 8). This means that this new formula was able to keep the activity of telomerase at a low level and therefore prevent the carcinogenesis of stem cells.

### 2.9. Telomere Length Assay

**Figure 9:**
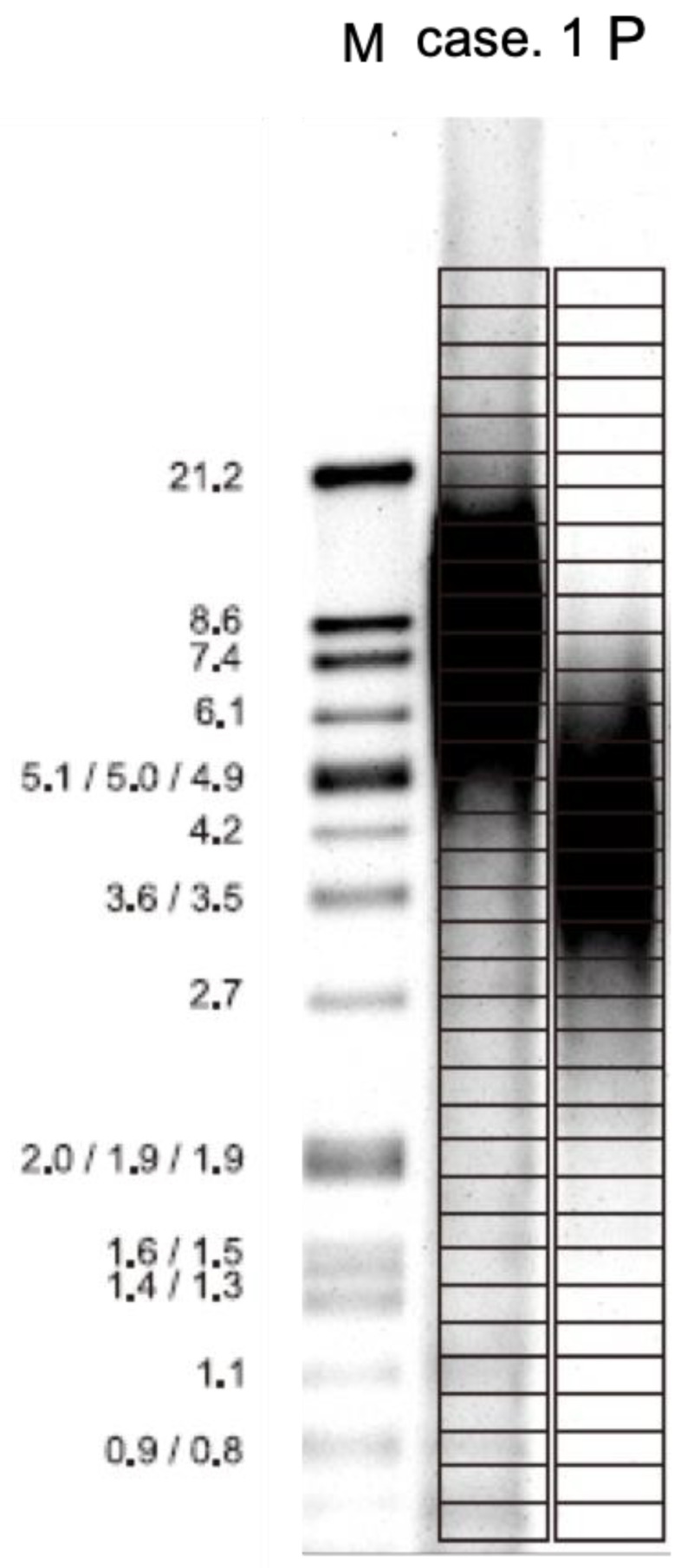
Telomerase length assay. Under the culture of this new formula, the HBMSCs of case 1 can maintain their length of telomere of 8 Kb, which is nearly the same as the length of the cells in newborns. M: marker, P: positive control.

The length of telomere decreased with the aging of cells. Under the culture of this new formula, the 4th passage of HBMSCs can maintain their length of telomere of 8 Kb (Figure 9), which is nearly the same as the length of the cells in newborns. Our new formula can maintain the length of the telomere, which can decrease the aging of stem cells.

## 3. Discussion

In this paper, we report a new formula for isolating and culturing HBMSCs that belong to adult stem cells and are rare in the bone marrow [26]. It has been discovered that particular cell culture media formulations and methods are useful in isolating and propagating HBMSCs due to their rarity. Because HBMSCs can proliferate without limitation and can contribute to at least three cell types, they offer a hope for tissue engineering; however, but adult HBMSCs in bone marrow are limited and not easy to amplify. At the same time, to keep the genetic and epigenetic characters unchanged during prolonged periods of culture, developing and maintaining a steady stage of these cells is still a challenge. An optimizing medium to reduce the rate of which genetic and epigenetic changes accumulate in culture is necessary. This paper identifies novel methods containing all compositions, to grow and propagate adult HBMSCs, and to keep those cells in a steady stage for a chance to differentiate. The ideal HBMSC culture medium should be: (a) easy for all researchers to use HBMSC as a study tool; (b) easy to maintain the characteristics of those cells as their human origin; (c) easy to grow and triggered for differentiation; (d) keep a steady stage as their human origin to reduce the genetic and epigenetic changes that accumulate in cultures. The HBMSCs cultured in our new formula meet all of the above requirements.

In previous studies, it was found that it was not easy for HBMSCs to amplify to passage 6—about 30 accumulated population doubling [1]. We modified culture media from MCDB153, like DMEM, with high concentration of calcium ion about 1.8 mM, to make a medium low calcium buffer media. HBMSCs differentiate when cultured in a high calcium medium, and the shape of the cells become flat. Cells cultivated early in low calcium (0.09 mM) cultures displayed characteristics similar to those previously reported for multipotential stem cells [22]. This cell culture medium includes an antioxidant agent vitamin C to maintain cells in a steady stage. The vitamin C may be provided in any form, but L-ascorbic acid-2 –phosphate (LS2P) is more efficient. Another antioxidant, N-acetyl-L-cysteine, was added to this media. N-acetyl-L-cysteine is both a powerful antioxidant and a precursor of L-cysteine, which promotes intracellular glutathione synthesis. It also showed that N-acetyl-L-cysteine significantly lowered levels of ROS in a short time on mesenchymal stem cell chondrogenesis [27]. The human serum used at the start of the culture offers an efficient replacement for continuous FCS treatment [28], but it involves high costs and has ethical issues that need to be considered. Particularly for this useful embodiment, the cell culture medium includes about 1 mM N-acetyl-L-cysteine, 5% FBS, trace ethanolamine, phosphoethanolamine, hydrocortisone, EGF, and bovine pituitary extract (BPE). This modified formula keeps HBMSCs in a steady stage, which can be long-term cultured and easy to be triggered to differentiate into osteocytes, adipocytes, and chondrocytes.

The common method to verify the isolated HBMSCs as stem cells is to examine the ability of multiple differentiations. Isolated HBMSCs at the 3rd, 6th, 13th, and 15th passages were easy to trigger for differentiation and expressed ECM calcification deposited stain with either von Kossa (black) or alizarin red S (red) under osteogenic differentiation—specific oil in adipocyte by oil red O (red) stain under adipogenic differentiation and glycoaminoglycan by alcian blue stain (blue) under chondrogeneic differentiation. The other way to verify the characters of HBMSCs is cell surface markers. HBMSCs must express CD29, CD44, CD73, CD90, CD105, and CD106 and should not express the hematopoietic lineage markers, including c-kit, CD14, CD11b, CD31, CD34, CD45, CD49D, CD56 CD62e, CD19, CD79α, CD106, CD133, and human leukocyte antigen (HLA)-DR [25]. Our results showed cultured HBMSCs at the 6th, 13th, and 15th passage expressed similar surface markers. All markers were specific to stem cells and none were specific to hematopoietic stem cells. This result is identical to that of previous studies.

The results also showed the same CGH array and 53BP1 DNA damage protein expression between the 5th and 15th passage culture of HBMSCs in this new formulation. They imply that this formula can prevent the accumulation of damage of chromosome and DNA. Besides, the length of the telomere can be maintained in this new formula without enhancing the activity of the telomerase. Senescence can be minimized under this new formulation and carcinogenesis of stem cells can also be prevented.

In conclusion, our new formula keeps HBMSCs in the state they were originally cultured in, with high proliferation rate and multiple linage differentiation ability offering a stable chromosome, DNA, telomere length, and telomerase activity even after long-term culture. This modification in culture medium can stabilize HBMSCs in long-term culture and the formula can be of great help to stem cell researchers.

## 4. Materials and Methods

### 4.1. Isolation of Human Bone Marrow Stroma Cells

The protocol for this study was approved by the institutional review board of Kaohsiung Medical University Hospital. Human bone marrow stroma cells were isolated from volunteer subjects with informed consent [20]. We excluded patients with liver or renal function insufficiency, hormone disorders, diabetes mellitus, pregnancy, and a past-history of neurological impairment or those who had taken glucocorticoid medications. Briefly, we aspirated 5 mL bone marrow from the iliac crest. The cells were separated by a Percoll™ (Amersham Pharmacia, Piscataway, NJ, USA) gradient; the nucleated stroma cells were collected for primary cell cultures [29]. The cells were maintained in Dulbecco modified Eagle medium (DMEM) (GibcoBRL, Gaithersburg, MD, USA) containing 10% fetal bovine serum (Hyclone Laboratories, Logan, UT, USA), fifty milligrams of sodium ascorbate per milliliter, and antibiotics (100 units of penicillin G per milliliter and 100 micrograms of streptomycin per milliliter) in a humidified atmosphere of 5% carbon dioxide at 37 °C. The media were changed every other day. After 15 days, the HBMSCs were attached and about 50% confluence, subcultured and seeded on BK media, as passenger 2, P2 culture. The medium, BK media, used to develop the human bone marrow stroma cells, were modified MCDB 153 media (Keratinocyte-SFM, GIBCO–Invitrogen) with medial calcium concentration 0.95 mM supplemented with *N*-acetyl-L-cysteine (NAC; Sigma, St Louis, MO, USA) (1 mM) and L-ascorbic acid 2-phosphate (Asc 2P; Sigma-Aldrich, St. Louis, MO, USA) (0.1 mM). The growth factors and hormones for this medium were recombinant epidermal growth factor (rEGF, 2.5 ng/mL; GibcoBRL, Gaithersburg, MD, USA), bovine pituitary extract (BPE, 25 μg/mL; GibcoBRL, Gaithersburg, MD, USA), insulin (2.5 μg/mL), hydrocortisone (37 ng/mL), and NAC (N- acetyl-cystin; Sigma-Aldrich, St. Louis, MO, USA). NAC is an antioxidant previously found to promote the self-renewal of stem cells [30] and is readily deacetylated in antioxidant cells to yield L-cysteine, thereby promoting the synthesis of reduced intracellular glutathione [31]. Ascorbic acid 2- phosphate is an effective means for providing ascorbic acid in cell culture [32,33].

### 4.2. Cumulative Population Doubling Level (cpdl)

Primary cell culture of HBMSCs were isolated and cultured as P1. The cells were cultured in BK medium or DMEM medium until 80% confluence, and then subcultured and seeded to a new dish for passages 2, P2. The passage was to analogize. Cumulative population doubling level (cpdl) in continual subculture and growth from a known number of cells was calculated to determine the proliferation potential of putative HBMSCs. The cpdl at each subcultivation was calculated from the cell count by using the following equation—ln (*Nf/Ni*)/ln2—where *Ni* and *Nf* are initial and final cell numbers, respectively, and ln is the natural log (ln).

### 4.3. Anchorage Independent Growth (AIG)

In vitro transformed cells are able to survive and grow in the absence of anchorage to the extracellular matrix (ECM) and their neighboring cells, termed anchorage independence of growth. We used a soft agar assay for colony formation as AIG. First, we prepared the 0.5% base agar layer in a 6-cm dish. Then a total of 50,000 cells in 3 mL of 0.33% ag [34] arose with BK medium were plated on top (as top agar layer) with a base layer agar. Then, 2.5 mL BK media was added to the dish and BM medium was changed every 2 days. The HBMSCs were cultured for 21 days, and the number of AIG colonies developed were counted under a microscope.

### 4.4. Multilineage Differentiation (Adipogenesis, Chondrogenesis, and Osteogenesis)

#### 4.4.1. Osteogenic Differentiation

To induce osteogenic differentiation, cells of the 5th to the 19th passage were treated with osteogenic medium for 12 days. The differentiation medium was changed every 2 days. Osteogenesis was assessed at weekly intervals. The osteogenic medium consisted of IMDM supplemented with 0.01 μM dexamethasone (Sigma-Aldrich, St Louis, MO, USA), 50μM glycerol phosphate (Sigma-Aldrich, St. Louis, MO, USA), and 0.2 mM ascorbic-2-phosphate (As2P; Sigma-Aldrich, St. Louis, MO, USA) [20,35,36,37,38,39,40,41,42,43,44,45,46,47,48,49,50,51,52,53,54,55,56]. The osteogenic differentiation of HBMSs was detected by Alizarin red staining. Briefly, the cells were fixed with 4% paraformaldehyde at room temperature for 10 min. After washing once with ddH_2_O, 0.5 mL alizarin red S solution (2%, pH 4.2) was added to each well in a 24-well plate. The staining solution was removed after 10 min. Each well was washed with ddH_2_O twice. The plate was then air-dried at room temperature and mineralization was detected by microscopy.

#### 4.4.2. Chondrogenic Differentiation

To induce chondrogenic differentiation, cells of the 5th to 19th passage were transferred into a 15-mL polypropylene tube and centrifuged at 1000 rpm for 5 min. After removing the supernatant, one million cells were moved into 10 μL media to form a pelleted micromass at a dish and treated with chondrogenic medium for 12 day. The medium was changed every 2 days and chondrogenesis was assessed at weekly intervals. The chondrogenic medium consisted of low-glucose DMEM (Gibco, Carlsbad, CA, USA) supplemented with TGF-1 (Sigma-Aldrich, St. Louis, MO, USA) 10ng/mL, L-Ascorbate -2-phosphate (Sigma-Aldrich, St. Louis, MO, USA) 50M, Insulin (Sigma-Aldrich, St. Louis, MO, USA) 6.25g/mL. The cells were stained with Alcian blue 8-GX overnight [57,58,59,60,61,62,63,64,65] to detect the chondrogenic differentiation.

#### 4.4.3. Adipogenic Differentiation

To induce adipogenic differentiation, cells of the 5th to the 19th passages were treated with adipogenic medium for 12 days. The medium was changed every other day and adipogenesis was assessed at weekly intervals. The adipogenic medium consisted of DMEM supplemented with 0.5 mM 3-isobutyl-1-methylxanthine (IBMX; Sigma-Aldrich, St. Louis, MO, USA), 1 μM Dexamethasone (Sigma-Aldrich, St. Louis, MO, USA), and 10 μg/mL insulin. Oil red O staining was used to detect adipogenic differentiation. Cells were fixed with 4% paraformaldehyde at room temperature for 10 min. After washing once with ddH_2_O, they were rinsed with 60% isopropanol. 0.5 mL oil red O solution (0.5% in isopropanol; Sigma-Aldrich, St. Louis, MO, USA) was added to each well in a 24-well plate for 15 min. Each well was rinsed with 60% isopropanol. The plate was then air-dried at room temperature and the adipogenic differentiation was detected by microscopy [46,66].

### 4.5. Flow Cytometry and Cell Surface Marker for Stem Cell Specificity

The cells were analyzed by flow cytometry using antibodies described in Table 1. HBMSCs were cultured in control medium 72 h before the analysis. Aliquots containing 5 × 10^5^ cells were incubated with primary antibodies. Flow cytometry with a FAC scan argon laser cytometer (BD, Biosciences, San Jose, CA, USA) was performed according to a previous study [57]. Briefly, the cells were harvested in 0.25% trypsin/EDTA and fixed for 30 min in ice-cold 70% EtOH. The fixed cells were washed in flow cytometry buffer (PBS, 2% FBS, 0.2% Tween 20) and incubated for 30 min in flow cytometry buffer containing fluorescein isothiocyanate-conjugated monoclonal antibodies CD antigens CD29, CD31, CD34, CD44, CD45, CD49d, CD56, CD62e, CD90, CD105, CD106, CD133, and CD166 to check for specific stem cell surface markers. HBMSCs were stained with a phycoerythrin-conjugated nonspecific IgG to assess background fluorescence [67]. Cell surface marker expression was determined by comparison with an isotype control on a histogram plot.

### 4.6. Array Comparative Genomic Hybridization (Array-CGH)

Molecular karyotyping was done through array comparative genomic hybridization (array-CGH) with the Agilent kit (Human Genome CGH Microarray, Agilent Technologies, Santa Clara, CA, USA). The array-CGH platform is a 60-mer oligonucleotide-based microarray that allows a genome-wide survey and molecular profiling of genomic aberrations with a resolution of f75 kb (kit 44B). The genetic situation of the 10 BM donors was tested before culture (defined as time 0 or T0), using bone marrow stroma cell and after in vitro culture on HBMSCs at P5 (the passage at which HBMSCs are usually harvested for the test; the accumulation doubling number is about 15 doubling). Three HBMSCs samples were also evaluated at later passages, between P15 (T2), after prolonged in vitro culture.

### 4.7. DNA Damage Protein 53BP1 Immunostaining

Cells were grown in BK medium supplemented with 5% fetal bovine serum at 37 °C with 5% CO_2_. UV light was delivered in a single pulse (50 J/m^2^) using a Stratalinker UV source (Stratagene, La Jolla, CA, USA) for positive control. Before UV irradiation, the culture medium was removed and replaced immediately after irradiation. All cells were returned to the incubator for recovery and harvested at the indicated times. The cells were harvested and fixed on 10% formalin in PBS for 30 min, blocked under 3% fetal bromine serum, stained with Rabbit polyclonal anti-53BP1 (Chemicon; Thermo Fisher Scientific, Waltham, MA, USA) for 1 hour at room temperature, and then stained with a secondary antibody. The Rabbit specific HRP/DAB (ABC) detection IHC kit (ab24621, Abcam; Cambridge, UK) was used to detect the primary antibody. The immuno-stains were examined by confocal microscopy.

### 4.8. Telomerase Activity Assay

About 3 × 10^7^ of HBMSCs at P5 and P15 were harvested for telomerase activity assay by the TeloTAGGG Telomerase PCR ELISA assay kit (Merck; Darmstadt, Germany). The cells that contained telomerase added these repetitive sequences to the 3’-end of the biotinylated synthetic P1-TS-primer (primer and nucleotides in reaction mixture, bottle 2). In a second step, these elongated products were amplified by PCR using primers P1-TS and P2, generating PCR amplicons. The telomerase PCR ELISA combined both reactions in a one-step/one-tube reaction. Optimized primer sequences eliminated the need for hot start PCR or the use of a wax barrier, avoiding amplification artifacts, such as primer dimers. An aliquot of the PCR was denatured and hybridized to a digoxigenin (DIG)-labeled, telomeric repeat-specific detection probe. The hybridization products were immobilized via the biotin-labeled primer to a streptavidin-coated microplate. The immobilized PCR product was detected with an anti-digoxigenin antibody conjugated to peroxidase. The probe was finally visualized by peroxidase, which metabolized TMB to form a colored reaction product. As an alternative to the ELISA protocol, the biotinylated primers were used for detection. If the telomerase-mediated six nucleotide incremental ladder was desired, fragments were separated by PAGE, blotted onto a positively charged membrane, and then detected appropriately (Biotin Luminescent Detection Kit).

### 4.9. Telomere Length Assay

Nonradioactive chemiluminescent assay was used to determine telomere length. HBMSCs cells were harvested and extracted for genomic DNA digested by an optimized mixture of frequently cutting restriction enzymes. The enzymes have been selected in such a way that telomeric DNA and sub-telomeric DNA were not cut. This was due to the special sequence characteristics of the repeats. Non-telomeric DNA was digested to low molecular-weight fragments. Following DNA digestion, the DNA fragments were separated by gel electrophoresis, then transferred to a nylon membrane by Southern blotting. The blotted DNA fragments were hybridized to a DIG-labeled probe that is specific for telomeric repeats, then incubated with a DIG-specific antibody covalently coupled to alkaline phosphatase. Finally, the immobilized telomere probe was visualized by a highly sensitive chemiluminescent substrate for alkaline phosphatase, CDP-Star. The average TRF length can be determined by comparing the signals to a molecular-weight standard; all images were detected by LEVEL BP-100, a Bio-Lab image system.

## 5. Conclusions

In conclusion, our new formula keeps HBMSCs in the state they were originally cultured in, with high proliferation rate and multiple linage differentiation ability offering a stable chromosome, DNA, telomere length, and telomerase activity, even after long-term culture. This new formula will be of great help for stem cell researchers.

## Figures and Tables

**Table 1:** The CD markers expressed on h-ASC, HBMSCs, osteoblast-like cells, HBMSC in traditional medium at P6 stage, BK medium at P6, P13, and P15 stages.

	h-ASC	BM-MSC	Osteoblast-Like Cells	BM-MSC Bone Media P6	BM-MSC P6 New Media	BM-MSC P13 New Media	BM-MSC P15 New Media
**CD 29**	+	+	+	+	+	+	+
**CD 31**	−	−	+	−	−	−	−
**CD 34**	+/−	−	+	−	−	−	−
**CD 44**	+	+				+	+
**CD 45**	−	−	−	−	−	−	−
**CD 56**	−	−	+	+/−	−	−	−
**CD 62e**	−	−	+	+/−	−	−	−
**CD 90**	+	+		+	+	+	+
**CD105**	+	+		+	+	+	+
**CD106**	−	−	−	−	−	−	−
**CD133**	−	−		+/−	+/−	−	−
**CD166**	+	+	+	+/−	+/−	+	+

## Data Availability

None.
